# The Role of Reactive Oxygen Species in β-Adrenergic Signaling in Cardiomyocytes from Mice with the Metabolic Syndrome

**DOI:** 10.1371/journal.pone.0167090

**Published:** 2016-12-01

**Authors:** Monica Llano-Diez, Jon Sinclair, Takashi Yamada, Mei Zong, Jeremy Fauconnier, Shi-Jin Zhang, Abram Katz, Kent Jardemark, Håkan Westerblad, Daniel C. Andersson, Johanna T. Lanner

**Affiliations:** 1 Karolinska Institutet, Department of Physiology & Pharmacology, Stockholm, Sweden; 2 Karolinska University Hospital, Rheumatology unit, CMM, Stockholm Sweden; 3 Karolinska Institutet, Department of Medicine, Stockholm, Sweden; Universidad Pablo de Olavide, SPAIN

## Abstract

The metabolic syndrome is associated with prolonged stress and hyperactivity of the sympathetic nervous system and afflicted subjects are prone to develop cardiovascular disease. Under normal conditions, the cardiomyocyte response to acute β-adrenergic stimulation partly depends on increased production of reactive oxygen species (ROS). Here we investigated the interplay between beta-adrenergic signaling, ROS and cardiac contractility using freshly isolated cardiomyocytes and whole hearts from two mouse models with the metabolic syndrome (high-fat diet and *ob/ob* mice). We hypothesized that cardiomyocytes of mice with the metabolic syndrome would experience excessive ROS levels that trigger cellular dysfunctions. Fluorescent dyes and confocal microscopy were used to assess mitochondrial ROS production, cellular Ca^2+^ handling and contractile function in freshly isolated adult cardiomyocytes. Immunofluorescence, western blot and enzyme assay were used to study protein biochemistry. Unexpectedly, our results point towards decreased cardiac ROS signaling in a stable, chronic phase of the metabolic syndrome because: β-adrenergic-induced increases in the amplitude of intracellular Ca^2+^ signals were insensitive to antioxidant treatment; mitochondrial ROS production showed decreased basal rate and smaller response to β-adrenergic stimulation. Moreover, control hearts and hearts with the metabolic syndrome showed similar basal levels of ROS-mediated protein modification, but only control hearts showed increases after β-adrenergic stimulation. In conclusion, in contrast to the situation in control hearts, the cardiomyocyte response to acute β-adrenergic stimulation does not involve increased mitochondrial ROS production in a stable, chronic phase of the metabolic syndrome. This can be seen as a beneficial adaptation to prevent excessive ROS levels.

## Introduction

The metabolic syndrome is a major and rapidly increasing health problem that includes obesity, insulin resistance, and type 2-diabetes. Patients with the metabolic syndrome are prone to cardiovascular disease, which is the most common cause of death in the Western world. Thus, elucidating the regulation of cardiac function and dysfunction in the metabolic syndrome is of fundamental importance [1]. The sympathetic nervous system is activated when the body is subjected to stress. The heart is then a key target and acute adrenergic activation of cardiomyocytes results in increased contractile force and faster relaxation, which are essential responses in situations with acute stress that depend on altered cellular Ca^2+^ handling. Conversely, prolonged hyperactivity of the sympathetic nervous system is associated with cardiac pathology, including impaired contractility that eventually might lead to heart failure and sudden cardiac death [2, 3].

Excessive levels of free radicals (i.e. reactive oxygen/nitrogen species (ROS)) are considered to have numerous deleterious effects leading to severe dysfunctions and disorders. In the heart, increased ROS levels have been associated with cardiac dysfunction, e.g. causing altered influx and refilling of Ca^2+^ stores, as well as left ventricular remodelling and heart failure [4–10]. However, we recently showed that increased mitochondrial ROS production plays an important role in the acute response of mouse cardiomyocytes to β-adrenergic stimulation [11] and similar results were subsequently obtained in rabbit cardiomyocytes [12].

Cardiac dysfunction occurs in patients with the metabolic syndrome [13–15] and the metabolic syndrome is accompanied by increased stress signaling, including signaling emanating from the sympathetic nervous system and changes in mitochondrial ROS signaling [16, 17]. The aim of this project was to characterize the inter-relationship between adrenergic stimulation and ROS in the control of Ca^2+^ handling and contraction in cardiomyocytes in the metabolic syndrome. Two mouse models of the metabolic syndrome were used: the genetically leptin-deficient *ob*/*ob* mice and a physiological approach with high-fat diet (HFD) [18, 19]. The effects of β-adrenergic stimulation on mitochondrial ROS production, cellular Ca^2+^ handling and contractile function were studied in freshly isolated adult cardiomyocytes. We hypothesized that cardiomyocytes of mice with metabolic syndrome would exhibit severe oxidative stress, impaired Ca^2+^ handling and contractility, and defective response to β-adrenergic stimulation. However, we found major adaptions to the prolonged stress resulting in decreased ROS production and spared functionality. Thus, in contrast to the situation in control cardiomyocytes, the acute response of metabolic syndrome cardiomyocytes to β-adrenergic stimulation was ROS-independent.

## Materials and Methods

### Ethical approval

All experiments complied with the Swedish Animal Welfare Act, the Swedish Welfare Ordinance, and applicable regulations and recommendations from Swedish authorities. The study was approved by the Stockholm North Ethical Committee on Animal Experiments (Permit Number: N120/13).

### Animals

Six weeks old C57bl/6N (6N) mice (Taconic, Denmark) were fed high-fat (45% fat of total calorie, Research Diets) diet (HFD) for 8 weeks, which results in obesity and insulin resistance [19]. Lean 6N mice fed control diet (10% fat of total calorie, Research Diets) were used as controls (Ctrl). In some experiments 8–10 weeks old 6N leptin-deficient, genetically obese male mice (*ob*/*ob*, Taconic, Denmark) and their wild-type (WT) littermates were used [18]. Varying fasting glucose levels have been reported (~4.5–8.5 mM) in control, HFD and *ob/ob* mice [18, 20–24]. The specific focus of the present study was on the effects of β-adrenergic stimulation on mitochondrial ROS production, cellular Ca^2+^ handling and contractile function and we therefore decided to keep glucose concentration of the superfusing Tyrode solution standardized at 5.5 mM (see detailed composition below). Mice were killed by rapid neck disarticulation, and the heart was excised. In vitro studies were performed at room temperature (20–24°C).

Nicotinamide nucleotide transhydrogenase (NNT) is an inner mitochondrial membrane protein, which can act as an antioxidant enzyme [25]. The mice in the present study were on the 6N background, which has functional NNT [25]. On the other hand, the C57bl/6J mouse strain has spontaneous mutations in the gene for NNT resulting in markedly decreased gene expression and this is accompanied by glucose intolerance [26]. The role of NNT in relation to cardiomyocyte ROS balance is complex and a recent study showed more oxidative damage in hearts from mice with 6N than with 6J background in a model of heart failure [25]. Thus, the difference in NNT activity between mice with 6N and 6J background has to be taken into account when interpreting results of ROS-dependent processes.

### DXA measurements

Body composition was measured using dual X-ray absorptiometry whole-body scan according to the manufacturer's instructions (DXA, Lunar Prodigy, GE Healthcare). Data were analyzed (head excluded) with the GE Encore 12.30 software.

### Glucose uptake

2-deoxyglucose (2-DG) uptake was measured in whole EDL and soleus muscles as previously described [27].

### Cryosectioning and oil red O staining

Whole hearts were frozen in isopentane precooled in liquid nitrogen and then cut into 7 μm thick sections and embedded on chrome gelatin-coated glass slides. Slides were air-dried and then fixed in 2% cold formaldehyde for 20 minutes and thereafter washed in cold phosphate-buffered saline (PBS). Oil red O (ORO) staining and analysis were performed as previously described [27]. Images of lipid accumulation were analyzed with Fiji (ImageJ).

### Cardiomyocyte isolation and measurement of cytosolic Ca^2+^

Single cardiomyocytes were isolated from the ventricles of mouse hearts and used within 3 hours as previously described [28]. Cardiomyocytes were loaded with fluorescent indicator and put on laminin-coated coverslips. Cells were then superfused with standard Tyrode solution (in mM): 121 NaCl, 5 KCl, 1.8 CaCl_2_, 0.5 MgCl_2_, 0.4 NaH_2_PO_4_, 24 NaHCO_3_, 0.1 EDTA, and 5.5 glucose. Cardiomyocytes were stimulated by brief current pulses given at 1 Hz. The free cytosolic Ca^2+^ concentration ([Ca^2+^]_i_) was measured with the fluorescent Ca^2+^ indicator fluo-3 and confocal microscopy as described earlier [29]. Confocal images were obtained by line scanning along the long axis of the cell. To enable comparisons between cells, the fluo-3 fluorescence signal (F) during contraction was divided by the fluorescence immediately before a stimulation pulse was given (F_o_). Cells were exposed to the β-adrenergic agonist isoproterenol (ISO, 100 nM). In some experiments, the general antioxidant *N*-acetylcysteine (NAC, 5 mM) was used in the presence or absence of ISO.

### Measurements of mitochondrial ROS production

Changes in mitochondrial O_2_*- production were monitored using the fluorescent indicator MitoSOX Red (Invitrogen) and confocal microscopy as described previously [29]. Briefly, cardiomyocytes were loaded with MitoSOX Red (5 μM) for 30 min at room temperature, followed by washout. Cells were exposed to the β-adrenergic agonist (ISO; 100 nM). Confocal images were obtained after 10 min of 1 Hz stimulation in standard Tyrode solution and subsequently after an additional 5 min of 1 Hz stimulation in the presence of ISO. MitoSOX Red fluorescence was measured in the same region of the cell at each time point. The signal from each cell was normalized to that immediately before application of ISO. Changes in ROS induced by application of a high concentration of H_2_O_2_ (1 mM) were also monitored using MitoSOX Red. In these experiments the cells were electrically stimulated at 1 Hz for at least 10 min before H_2_O_2_ was applied and the fluorescence signal from each cell was normalized to that immediately before application of H_2_O_2_.

### Enzyme activities and mitochondrial ATP production

Left ventricles were homogenized in ice-cold buffer (50 *μ*l per mg wet weight) consisting of 50 mM KH_2_PO_4_, 1 mM EDTA and 0.05% Triton X-100, pH 7.5. The homogenate was centrifuged at 1400 *g* for 1 min at 4°C. The supernatant was used for analyses of citrate synthase (CS), *β*-hydroxyacyl-coenzyme A dehydrogenase (*β*-HAD) and glutathione S-transferase (GST) activities using standard spectrophotometric techniques [30, 31]. Activities were measured at room temperature under conditions that yield linearity with respect to extract volume and time. The protein content was determined using the Bradford assay and activities were adjusted for protein content. The total SOD activity was measured with a kit based on the reduction of tetrazolium salt induced by superoxide production (Cayman Chemicals) as described previously [32]. The mitochondrial ATP-production rate (MAPR) in isolated mitochondria was determined with a firefly luciferase method at 25°C by using a 1251 luminometer (BioOrbit Oy) as previously described [33]. MAPR was determined in the presence of palmitoyl-carnitine at four different concentrations and presented as the ATP synthesis rate (units) per unit of CS activity (MAPR/CS).

### Western blotting

Left ventricles were homogenized in ice-cold buffer (20 μl (mg wet weight)^-1^) consisting of (mm): 20 Hepes, 150 NaCl, 5 EDTA, 25 KF, 1 Na_3_VO_4_; 20% glycerol; 0.5% Triton X-100, and 1 tablet of protease inhibitor cocktail (Roche) per 50 ml, pH 7.4. Equal amounts of protein (5–15 μg) were separated by electrophoresis and transferred onto membranes as previously described [34]. The following antibodies were used: anti-phospholamban (PLB; 1:1000, Abcam), anti-PLB-phospho Ser^16^ (PLB-P Ser16; 1:2500, Badrilla), anti-β_1_-adrenergic receptor (AR; 1:1000, Abcam), anti-β_2_-AR (1:1000, Abcam), anti-superoxide dismutase (SOD) 1 (1:1000, Abcam) and 2 (1:1000, Upstate), anti-3-nitrotyrosine (3-NT; 1:500, Abcam), anti-maldialdehyde (MDA, 1:500, Abcam), anti-NAPH oxidase 2 (NOX2/gp91phox, 1:1000, Abcam), anti-NOX4 (1:2000, Thermo-Pierce). IRDyes (1:15,000, LI-COR), anti-GAPDH (1:1000, Abcam). In one set of experiments whole hearts were perfused with Tyrode solution for 20 min in the presence or absence of ISO (100 nmol/l) or H_2_O_2_ (1 mmol/l) using a Langendorff setup. Left ventricles were then snap-frozen and analyzed for MDA protein adducts.

### Immunofluorescence

Immunofluorescence on freshly isolated cardiomyocytes was performed as described previously [18]. Cells were then incubated with anti-ryanodine receptor 2 (anti-RyR2, Abcam) and anti-β_1_-AR or anti-β_2_-AR antibodies (1:50 dilution in 1% BSA) at 4°C overnight and then washed and incubated with fluorescent secondary antibodies (Alexa Fluor 488 and 594, Invitrogen). Images of longitudinal thin sections of stained cells were obtained with laser confocal microscopy with a Nikon Plan Apo 100x oil immersion objective. Focus was set at the height where the cell diameter was maximal.

### L-type Ca^2+^ current measurements

Whole-cell patch-clamp recordings were performed on freshly isolated cardiomyocytes as described previously [11], and a HEKA EPC9 amplifier was used for data acquisition. All data was sampled at 10kHz and filtered at 1kHz. Currents, recorded with 2–3 MΩ patch pipettes, were normalized to cell membrane capacitance and expressed as current densities (pA/pF). To record L-type Ca^2+^ currents, the following pipette solution was used (mM): 120 CsCl, 6.8 MgCl_2_, 5 Na_2_ATP, 5 sodium creatine phosphate, 0.4 Na_2_GTP, 11 EGTA, 4.7 CaCl_2_ (120 nM free [Ca^2+^]), and 20 HEPES; pH was adjusted with CsOH to 7.2. The bath solution contained (mM): 135 TEA-Cl, 2 MgCl2, 10 Glucose, 10 HEPES, 1.8 CaCl_2_; pH adjusted to 7.4 with TEAOH. For NAC experiments cells were pre-incubated for 10 minutes in buffer consisting of (mM): 135 NaCl, 20 NAC, 4 KCl, 2 MgCl_2_, 10 Glucose, 10 HEPES, 1.8 CaCl_2;_ pH adjusted to 7.4 with NaOH. The current-voltage (*I-V*) relationships were obtained by giving test pulses varying from -80 mV to +50 mV from a holding potential of -80 mV [35]. Electrophysiological data acquisition and analyses were performed using HEKA PatchMaster.

### Statistics

Student’s unpaired t-test was used for comparing two groups. One-way ANOVA was used to compare means of three groups and when this showed significant difference, the Holm-Sidak method *post hoc* test was performed. p<0.05 was considered significant. Data are expressed as mean ± SEM.

## Results

### The increased contractility in response to β-adrenergic stimulation is ROS-independent in metabolic syndrome cardiomyocytes

**Fig 1:**
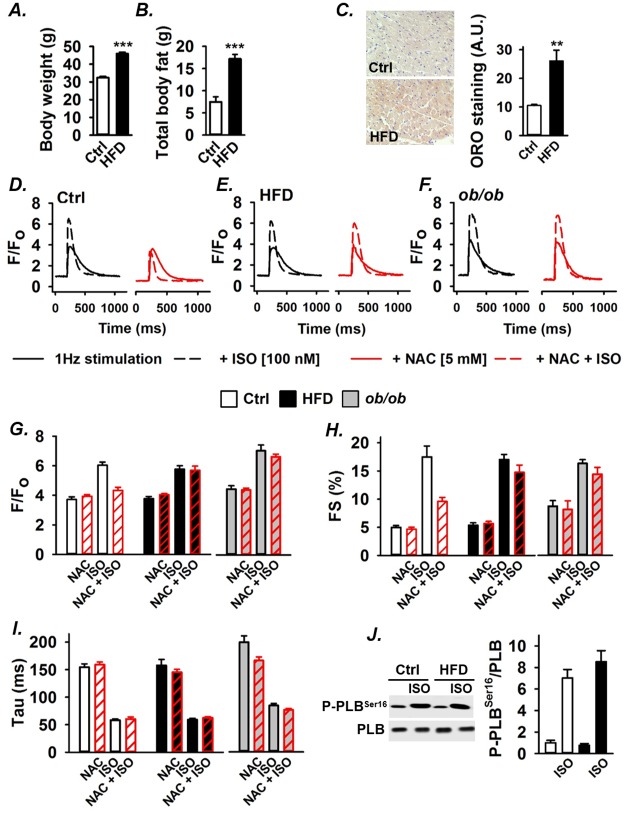
The antioxidant NAC has no effect on the β-adrenergic stimulated SR Ca^2+^ release and contractility in cardiomyocytes from mice with the metabolic syndrome. **(A)** Total body weight of mice after 8–10 weeks on control diet (Ctrl, n = 34) or high fat diet (HFD, n = 40). **(B)** Total body fat measured with DXA whole-body scan (n = 9–10). **(C)** Typical example of cross-sectional ORO staining showing fat accumulation (stained red) in left ventricles and mean data of ORO staining; eight sections per left ventricle from each group were analyzed (n = 3). Representative [Ca^2+^]_i_ transients from ctrl **(D)**, HFD **(E)** and *ob/ob* **(F)** cardiomyocytes obtained in the absence (full lines) and presence (dashed lines) of ISO (100 nM), and without (black lines) and with (red lines) NAC (5 mM). Average amplitude of Ca^2+^ transients **(G)**, fractional cell shortening (FS, ***H***) and [Ca^2+^]_i_ transient decay time constant (tau) **(I)** with ISO and/or NAC as indicated from control (white bars), HFD (black bars) and *ob/ob* (grey bars) cardiomyocytes (n>16 cells from at least three mice). Representative Western blots **(J)** and mean data (n = 6) of ISO-induced PLB phosphorylation normalized to total PLB expression in left ventricles from control and HFD mice. Data are mean ± SEM; **P < 0.01, ***P < 0.001.

β-adrenergic-induced increases in cardiomyocyte contractility and relaxation speed are traditionally considered to be the effect of cAMP-dependent protein kinase (PKA)-mediated phosphorylation of proteins involved in cardiomyocyte Ca^2+^ handling. However, we recently showed that the stimulatory contractile effects of β-adrenergic stimulation with ISO partly depend on increased mitochondrial ROS production in mouse WT cardiomyocytes [11] and similar results were subsequently obtained in rabbit cardiomyocytes [12]. The metabolic syndrome is associated with a prolonged increased sympathetic drive [15, 17], which might result in excessive ROS production and deleterious oxidative stress. Here we studied the relationship between β-adrenergic stress, ROS and contractility in cardiomyocytes from mice with the metabolic syndrome (HFD and *ob/ob* mice). After 8–10 weeks, mice on HFD had gained ~ 40% more weight than their lean control counterparts (Fig 1*A*) and DXA measurements showed that this was due to a large increase in fat mass (Fig 1*B*). HFD mice also exhibit peripheral insulin-resistance (S1 Fig). ORO staining showed a markedly increased amount of fat in the heart: the abundance of lipid droplets was ~2.5 times higher in mice on HFD than in controls (Fig 1*C*). The *ob/ob* and WT mice had a body weight of ~50g and ~25 g, respectively. Typical [Ca^2+^]_i_ transients (Fig 1*D*–1*F*) and average data (Fig 1*G*–1*I*) show that ISO (100 nM) increased the [Ca^2+^]_i_ transient amplitude and decay rate as well as cell shortening in control, HFD and *ob*/*ob* cardiomyocytes paced at 1 Hz. The general antioxidant NAC (5mM) reduced ISO-induced increases of the [Ca^2+^]_i_ transient amplitude (Fig 1*G*, white bars) and cell shortening (Fig 1*H*, white bars) in control cardiomyocytes, whereas the rate of [Ca^2+^]_i_ decline remained unaffected (Fig 1*I*, white bars). In contrast, NAC did not affect the response to ISO in cardiomyocytes from mice on HFD (black bars) or *ob/ob* mice (grey bars). Thus, the β-adrenergic increase in SR Ca^2+^ release and contractility was mediated via ROS-dependent and ROS-independent signaling in control cardiomyocytes and in cardiomyocytes with the metabolic syndrome, respectively.

NAC did not affect the rate of [Ca^2+^]_i_ decline in either control or metabolic syndrome cardiomyocytes (Fig 1*I*). The increased [Ca^2+^]_i_ decay rate with β-adrenergic stimulation is considered to depend on PKA-mediated phosphorylation of phospholamban (Ser^16^) that leads to increased activity of the SR Ca^2+^-ATPase (SERCA) and hence accelerated SR Ca^2+^ uptake [36, 37]. We measured phopholamban protein expression and extent of ISO-stimulated phosphorylation in hearts from control and HFD mice. The results show no difference in phopholamban expression between the groups and ISO induced phospholamban Ser^16^ phosphorylation of a similar magnitude in control and HFD hearts (Fig 1*J*). Thus, these results indicate that the increased rate of [Ca^2+^]_i_ decline with β-adrenergic stimulation relied on ROS-independent phospholamban phosphorylation in both control and metabolic syndrome cardiomyocytes.

### No alteration in β_1_- or β_2_-AR distribution in cardiomyocytes from mice with the metabolic syndrome

**Fig 2:**
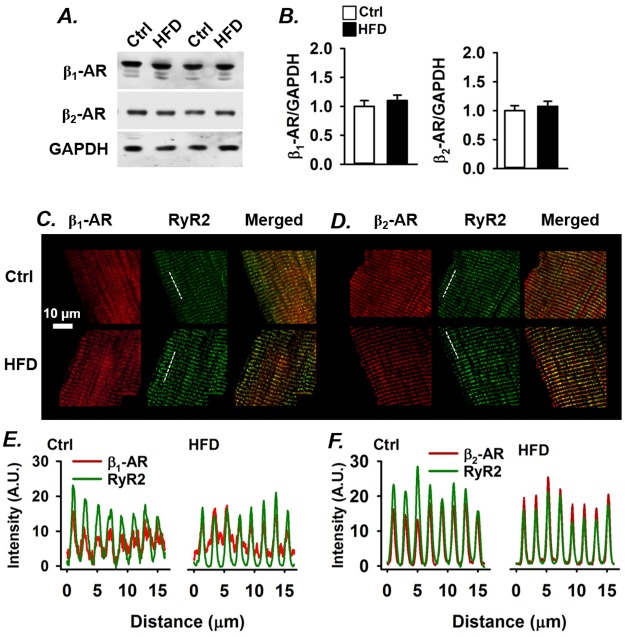
Cardiomyocytes from mice with the metabolic syndrome show normal β_1_-AR, β_2_-AR expression and distribution. **(A)** Representative Western blots and mean data ± SEM (***B***; n = 6) of total expression of β_1_- and β_2_-ARs in left ventricles from control mice and HFD mice. Immunofluorescence staining of β_1_-AR **(C)** and β_2_-AR **(D)** co-stained with RyR2 in control and HFD cardiomyocytes. Merged (yellow) show the intensity overlap between β-ARs and RyR2 in the dyads. ***E*** and ***F*** show plotted intensity profiles of along the dashed lines in *C* and *D* with β-AR (red) and RyR2 (green).

The difference in ROS dependency of the ISO-induced increase in SR Ca^2+^ release between control and metabolic syndrome cardiomyocytes (see Fig 1) might relate to differences in β_1_- and/or β_2_-AR expression or cellular distribution. For instance, remodelling of the t-tubular system and altered distribution of β-ARs have been observed in different models of heart failure [38, 39]. The total protein expression of β_1_- and β_2_-ARs was similar in control and HFD hearts (Fig 2*A* and 2*B*). Fig 2*C* and 2*D* show immunofluorescence images of the distribution of β_1_- and β_2_-ARs in cardiomyocytes from control and HFD mice. The SR Ca^2+^ release channel, RyR2, was used as a marker of the dyads, i.e. the regions where the t-tubules are in close connection to the SR. In accordance with previous publications from healthy cardiomyocytes [39], β_1_-ARs were distributed over the surface membrane and the t-tubular region (Fig 2*C* and 2*E*), whereas the β_2_-AR distribution was more restricted to the t-tubular region (Fig 2*D* and 2*F*). No differences were observed in the β_1_- and β_2_-AR distribution between control and HFD cardiomyocytes. Thus, the lack of a NAC effect on the ISO-induced stimulation of SR Ca^2+^ release in metabolic syndrome cardiomyocytes cannot be explained by altered expression or distribution of β_1_- and β_2_-ARs.

### The β-adrenergic-induced increase in Ca^2+^ influx via the L-type channel is ROS-independent in metabolic syndrome cardiomyocytes

**Fig 3:**
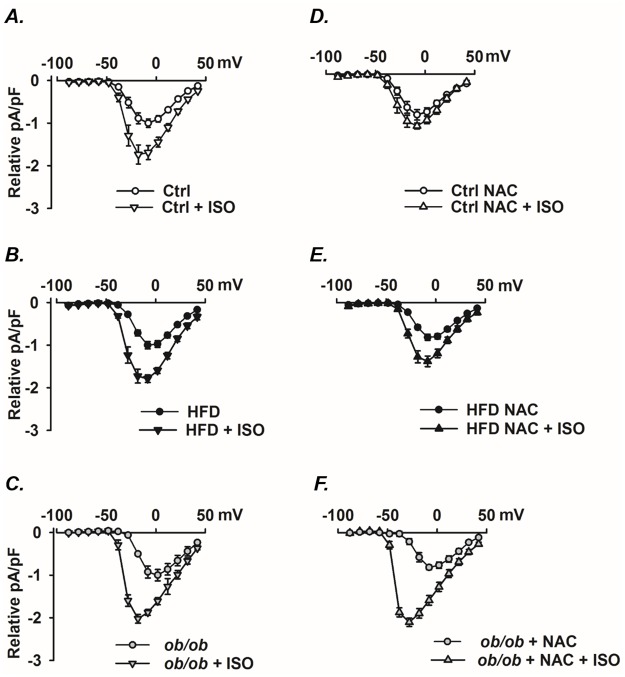
The ISO-induced increases in L-type Ca^2+^ current density is ROS-independent in cardiomyocytes with the metabolic syndrome. Mean data (±SEM; n = 6–8 in each group) of *I-V* curves of peak current density in the absence and presence of ISO (100nM) and NAC (20 mM) as indicated from control **(A, D)**, HFD **(B, E)** and *ob/ob* **(C, F)** cardiomyocytes. The *I-V* relationships were obtained by giving test pulses varying from -80 mV to +50 mV from a holding potential of -80 mV. The mean basal current density (i.e. in the absence of NAC and ISO) was -6.1±0.5 pA/pF and for comparisons between groups each group were normalized its basal current to give a relative current density (relative pA/pF).

In cardiomyocytes, the RyR2-mediated SR Ca^2+^ release is initiated by Ca^2+^ influx via the L-type channel (Cav1.2) located in the t-tubular system, i.e. Ca^2+^-induced Ca^2+^-release [40, 41]. ISO increased the L-type Ca^2+^ current in control, HFD and *ob/ob* cardiomyocytes (Fig 3*A*–3*C*). The ISO-induced increase in L-type Ca^2+^ current was blocked by NAC in control cardiomyocytes (Fig 3*D*), but not in HFD and *ob/ob* cardiomyocytes (Fig 3*E* and 3*F*). The β-adrenergic-induced L-type Ca^2+^ channel activation involves cAMP-PKA-dependent phosphorylation of the channel [42, 43]. Thus, the present results indicate that this signaling includes a ROS-dependent component in WT cardiomyocytes, which agrees with previous results from our group [11], whereas the signaling is ROS-independent in HFD and *ob*/*ob* cardiomyocytes.

### β-adrenergic stimulation does not increase mitochondrial ROS production in metabolic syndrome hearts

**Fig 4:**
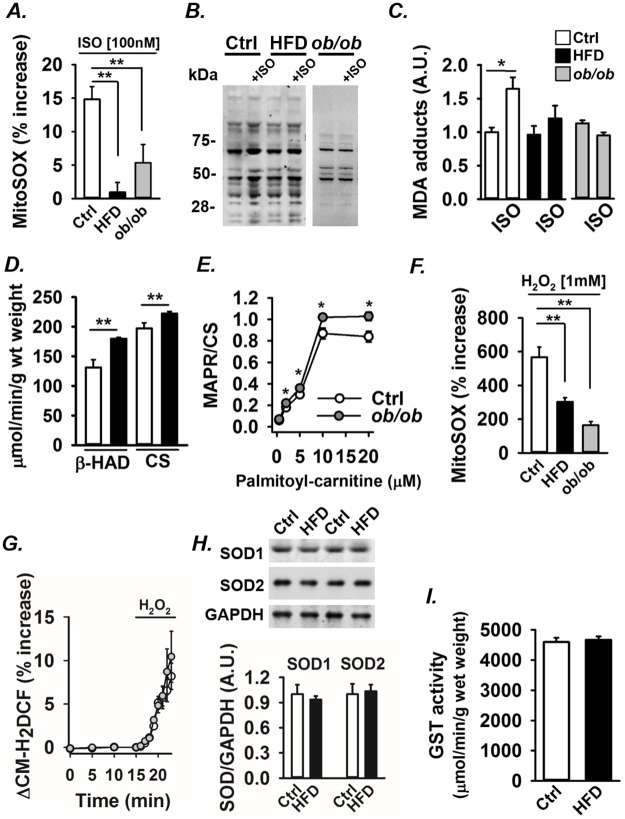
β-adrenergic stimulation increases mitochondrial ROS production in WT but not in metabolic syndrome hearts. **(A)** Mean data of mitochondrial ROS production in response to ISO (100 nM) measured with MitoSOX Red in WT (n = 29), HFD (n = 19) and *ob/ob* (n = 25) cardiomyocytes. Representative Western blots **(B)** and mean data **(C)** of MDA protein adducts in control, HFD and *ob/ob* hearts with or without ISO perfusion (100 nM) as indicated (n = 4–6 in each group; data in each group normalized to mean value in control cardiomyocytes without ISO, which was set to 1.0). **(D)** β-HAD and CS activity in left ventricles from control and HFD hearts (n = 6). **(E)** Mitochondrial ATP-production rate (MAPR) in isolated mitochondria determined in the presence of 0.5–20 μM palmitoyl-carnitine presented as the ATP synthesis rate is given per of CS activity (MAPR/CS; n = 6). **(F)** Mean data of mitochondrial ROS during application of H_2_O_2_ (1 mM) measured with MitoSOX Red in control (n = 45), HFD (n = 21) and *ob/ob* (n = 26) cardiomyocytes. **(G)** Mean data of cytosolic ROS, measured with the general ROS indicator CM-H_2_DCFDA, during exposure to H_2_O_2_ (1 mM) in control (n = 19) and *ob/ob* (n = 21) cardiomyocytes. Representative Western blots of SOD1 and SOD2 protein expression and mean data **(H)** in control and HFD left ventricles. (**I)** Mean data of the total GST activity in left ventricles from control and HFD hearts. Data are presented as mean ± SEM; *P < 0.05, **P < 0.01 *vs*. control (*A* and *D-F*); *P < 0.05 *vs*. without ISO (C).

We previously showed a cAMP-PKA-dependent increase in mitochondrial ROS production after ISO application [11]. Accordingly, ISO (100 nM) induced a ~15% increase in MitoSOX Red fluorescence in control cardiomyocytes (Fig 4*A*, white bar). Cardiomyocytes from HFD (black bar) and *ob/ob* (grey bar) mice showed markedly smaller increases in MitoSOX Red fluorescence in response to ISO application than control cells, which is consistent with the lack of NAC effects on cellular Ca^2+^ handling described above.

MDA adducts were used as a biomarker of ROS production. Whole hearts were perfused with Tyrode solution in a Langendorff setup for 20 min with or without ISO and then flash frozen and analyzed for MDA protein adducts. No ISO-induced increase in MDA protein adducts was observed in hearts from HFD and *ob/ob* mice, which contrasts to control hearts where ISO exposure resulted in an almost doubling of MDA protein adducts (Fig 4*B* and 4*C*). Conversely, the amount of MDA adducts in absence of ISO was similar in control and HFD hearts (Fig 4*C*) and similar results were obtained in control *vs*. *ob*/*ob* hearts (data not shown). Protein nitration was measured to further assess possible differences in baseline ROS levels and the results showed no difference in protein 3-NT levels between control and HFD hearts (S1 Fig). Thus, only control cardiomyocytes displayed increased ROS production in response to β-adrenergic stimulation and the lack of an ROS increase in HFD and *ob/ob* hearts was not explained by excessive ROS levels already under basal conditions.

A shift in metabolism towards fat utilization is a characteristic of hearts with the metabolic syndrome [44–46]. The activity of β-HAD, the rate limiting enzyme in fatty acid β-oxidation, was used as an indicator of β-oxidation efficiency in the hearts. β-HAD was ~40% higher in HFD than in control hearts (Fig 4*D*). Citrate synthase (CS) activity, used as a marker of mitochondrial content in cardiomytes, was ~15% higher in HFD than in control hearts (Fig 4*D*). Further, mitochondrial ATP-production rate (MAPR) was used to assess mitochondrial respiration. Isolated mitochondria from *ob/ob* hearts showed ~20% higher MAPR from palmitoyl-carnitine oxidation than mitochondria from control hearts (Fig 4*E*). Thus, mitochondria from HFD and *ob*/*ob* hearts showed a shift towards fat utilization and their lack of β-adrenergic-induced ROS production cannot be explained by an overall decrease in mitochondrial metabolism or oxidative phosphorylation.

### Decreased mitochondrial ROS production rather than increased ROS clearance in cardiomyocytes from mice with the metabolic syndrome

We previously reported that the saturated fatty acid palmitate markedly increases mitochondrial ROS production in cardiomyocytes from WT mice, but not from *ob/ob* mice [29]. Here we show that β-adrenergic stimulation increases mitochondrial ROS generation in control, but not in HFD and *ob*/*ob* cardiomyocytes. Therefore, we tested whether there was lower mitochondrial ROS production in general in HFD and *ob*/*ob* cardiomyocytes. The mitochondrial matrix contains manganese-dependent superoxide dismutase (MnSOD or SOD2), which converts superoxide ions (O_2_*-) to hydrogen peroxide (H_2_O_2_). To assess the basal rate of mitochondrial O_2_*- production, cardiomyocytes loaded with MitoSOX Red were paced at 1 Hz and then exposed to 1 mM H_2_O_2_, which will increase mitochondrial [O_2_*-] by inducing product inhibition of SOD2 and thereby inhibit the conversion of O_2_*- to H_2_O_2_ [47, 48]. H_2_O_2_ application resulted in an increase in the MitoSOX Red fluorescence of ~ 550% in control cardiomyocytes and the response was significantly smaller in HFD and *ob/ob* cardiomyocytes (Fig 4*F*). This difference depended on mitochondrial ROS production because application of H_2_O_2_ resulted in similar increases in fluorescence in control and *ob*/*ob* cardiomyocytes when experiments were performed with the cytosolic ROS indicator CM-H_2_DCF, which directly senses the externally applied H_2_O_2_ (Fig 4*G*).

NADPH oxidases (NOX) have received increasing attention as important cellular ROS producers [49, 50]. NOX2 and NOX4 are expressed in cardiomyocytes [51, 52]. We therefore measured the protein expression of NOX2 and NOX4 in control and HFD hearts but observed no difference between the two groups (S1 Fig).

Changes in mitochondrial ROS metabolism can be due to altered SOD2 activity [53]. We have previously shown similar SOD2 protein expression in hearts of WT and *ob/ob* mice [29]. Consistent with these findings, we observed no difference in SOD1 and SOD2 protein expression between control and HFD hearts (Fig 4*H*). Nor did we observe any significant difference in total SOD activity between the two groups (WT: 1169±17 U/mg, *ob/ob*: 1345±86 U/mg; n = 3, p > 0.05). Glutathione-S-transferase (GST) is another potent antioxidant enzyme that is responsible for inactivation of electrophilic compounds and toxic substrates [54]. Cytosolic GST enzyme activity was measured in control and HFD hearts and no significant difference was detected between the two groups (Fig 4*I*). To sum up, our results indicate that the decreased ROS levels in metabolic syndrome hearts is due to decreased mitochondrial ROS production rather than increased ROS clearance.

## Discussion

Traditionally, the metabolic syndrome has been associated with increased ROS-induced damage, which has been considered a contributor to diabetic cardiomyopathy [5, 8, 10]. However, here we demonstrate, with two different mouse models and several different methodological approaches, a consistently *decreased* ROS-induced signaling in cardiomyocytes of mice with the metabolic syndrome. Interestingly, decreased mitochondrial ROS production was recently also observed in kidneys from diabetic mice [55].

There are complex interactions between the rate of mitochondrial respiration and ROS production and these can be altered in mitochondria exposed to stress [56]. Previous studies have shown a role of mitochondrial ROS production in the β-adrenergic signaling in cardiomyocytes. For instance, application of the PKA catalytic subunit resulted in increased ROS production and altered the redox state in mitochondria of permeabilized rat cardiomyocytes [57]. Moreover, acute β-adrenergic stimulation with ISO resulted in a cAMP-PKA-dependent increase in mitochondrial ROS production in intact ventricular cardiomyocytes of WT mice [11]. This increase in ROS played an important role in the β-adrenergic inotropic effect, because the ISO-induced increases in [Ca^2+^]_i_ transient amplitude, contractility, and L-type Ca^2+^ current were diminished in the presence of the antioxidant NAC [11]. Subsequently Bovo *et al*. reported that β-adrenergic activation results in increased ROS production also in rabbit cardiomyocytes [12].

The present measurements of mitochondrial ROS production with MitoSOX Red show markedly smaller responses to β-adrenergic stimulation in HFD and *ob/ob* cardiomyocytes than in control cardiomyocytes (see Fig 4*A*). Measurements of MDA protein adducts are frequently used to assess increased ROS production [34, 58] and exposure to ISO resulted in a significant increase in MDA protein adducts in control, but not in HFD and *ob*/*ob* hearts (see Fig 4*B* and 4*C*). HFD and *ob*/*ob* cardiomyocytes responded to β-adrenergic stimulation with increases in [Ca^2+^]_i_ transient amplitude, cell shortening and L-type Ca^2+^ current but, in contrast to control cardiomyocytes, these responses were not inhibited by exposure to the antioxidant NAC (see Figs 1 and 3). On the other hand, β-adrenergic stimulation caused a NAC-independent increase in the [Ca^2+^]_i_ transient decay rate, which was accompanied by a similar increase in phospholamban Ser^16^ phosphorylation in control and HFD hearts (see Fig 1*J*). To sum up, β-adrenergic stimulation increases mitochondrial ROS production in control cardiomyocytes and in these the stimulatory effect on contractility involves both ROS-dependent and ROS-independent signaling. On the other hand, β-adrenergic stimulation does not increase mitochondrial ROS production in HFD and *ob*/*ob* cardiomyocytes; thus the stimulatory effect of ISO in these cells depends on ROS-independent signaling.

Our results with MitoSOX Red and H_2_O_2_ application indicate, if anything, a decreased basal rate of mitochondrial ROS production in HFD and *ob/ob* cardiomyocytes (see Fig 4*F*). Accordingly, there were no signs of increased basal oxidation in hearts of HFD and *ob/ob* mice, because hearts of control mice and mice with the metabolic syndrome showed similar levels of MDA protein adducts and protein nitration under basal conditions, similar protein expression of SOD1, SOD2, NOX2 and NOX4, and similar GST activity. However, it must be noted that the present HFD and *ob*/*ob* mice were in a stable, chronic phase (8 weeks of high-fat diet) and our findings do not exclude the possibility that oxidative stress occurs in earlier (i) and/or later (ii) phases or in alternative animal models of the metabolic syndrome (iii). In fact, such scenarios are supported by previous findings: i) Large increases in mitochondrial ROS production, accompanied by decreased [Ca^2+^]_i_ transients and contractility, were observed in cardiomyocytes of WT mice acutely exposed to the saturated fatty acid palmitate at a concentration commonly found in the blood from patients with the metabolic syndrome [29]. Intriguingly, the palmitate-induced increase in mitochondrial ROS production did not occur in *ob*/*ob* cardiomyocytes and their Ca^2+^ handling and contractility were, if anything, improved in the presence of palmitate [29]. Furthermore, administration of the antioxidant mitoTEMPO throughout 8 weeks of high-fat diet prevents insulin resistance and contractile alterations [59]. ii) Male c57bl/6 mice fed high-fat, high-sucrose diet for 8 months showed increased rate of H_2_O_2_ production, decreased glutathione activity and increased oxidative-induced posttranslational modifications [60]. iii) Many reports that refer to increased oxidative stress in the diabetic cardiomyopathy were performed with animal models where high-sugar diets were used [4, 61–64]. Furthermore, the increased insulin levels associated with insulin resistance may affect cardiac function: insulin has been shown to inhibit β-adrenergic stimulation and hence impair β-adrenergic-regulated cardiac contractility [65]; application of insulin increased the amplitude of [Ca^2+^]_i_ transients in wild-type cardiomyocytes, whereas it broadened the transients and triggered extra [Ca^2+^]_i_ transients in *ob*/*ob* cardiomyocytes [18]. Thus, the overall effects on cardiomyocyte function of the multiple alterations in extracellular environment and cellular signaling associated with the metabolic syndrome are highly complex. The present study was focused on the role of ROS in β-adrenergic signaling. The composition of the superfusing Tyrode solution was therefore kept constant with regards to other components that are changed *in vivo* in the metabolic syndrome (e.g. the concentration of glucose, fatty acid and insulin) and further studies are required to elucidate the interacting effects of multiple changes of the extracellular environment.

In conclusion, we propose the following scenario: In early states of the metabolic syndrome two types of systemic stress signals, increased β-adrenergic stimulation and elevated levels of saturated fatty acids, trigger mitochondrial ROS production in cardiomyocytes. This increased ROS production constitutes a serious challenge, which may result in severe pathological changes and contribute to the cardiac dysfunction often seen in patients with the metabolic syndrome [13–15]. Alternatively, the ROS increase results in major beneficial adaptations, which include a change in substrate utilization towards a preference for fatty acid oxidation [44–46]. Our present and previous [29] results show that cardiomyocytes of HFD and *ob/ob* mice follow the latter pathway reaching a state where β-adrenergic stimulation and elevated levels of saturated fatty acids no longer cause an increased mitochondrial ROS production.

## Supporting Information

S1 Fig **A.** Glucose uptake in fast-twitch (EDL) and slow-twitch (soleus) muscles shows that mice on fat diet have a decreased insulin-mediated glucose uptake, which verifies a systemic insulin resistance (n = 6). Data are mean ± SEM; * *P* < 0.05, ***P <0.001 *vs*. control). **B.** Representative Western blots and mean data ± SEM (n = 6) of protein nitration (anti-3-nitrotyrosine (3-NT; 1:500, Abcam)) in left ventricles from control mice and HFD mice. Representative Western blots **(C)** and mean data ± SEM (**D**, n = 6) of total expression of NOX2/gp91phox (1:1000, Abcam) and NOX4 (1:2000, Thermo-Pierce) in left ventricles from control mice and HFD mice.
(TIF)
