# Supplementary figures and images for: The Role of Reactive Oxygen Species in β-Adrenergic Signaling in Cardiomyocytes from Mice with the Metabolic Syndrome
Source: PLoS One. 2016 Dec 1;11(12):e0167090. doi: 10.1371/journal.pone.0167090 (PMC5131978; doi:10.1371/journal.pone.0167090)

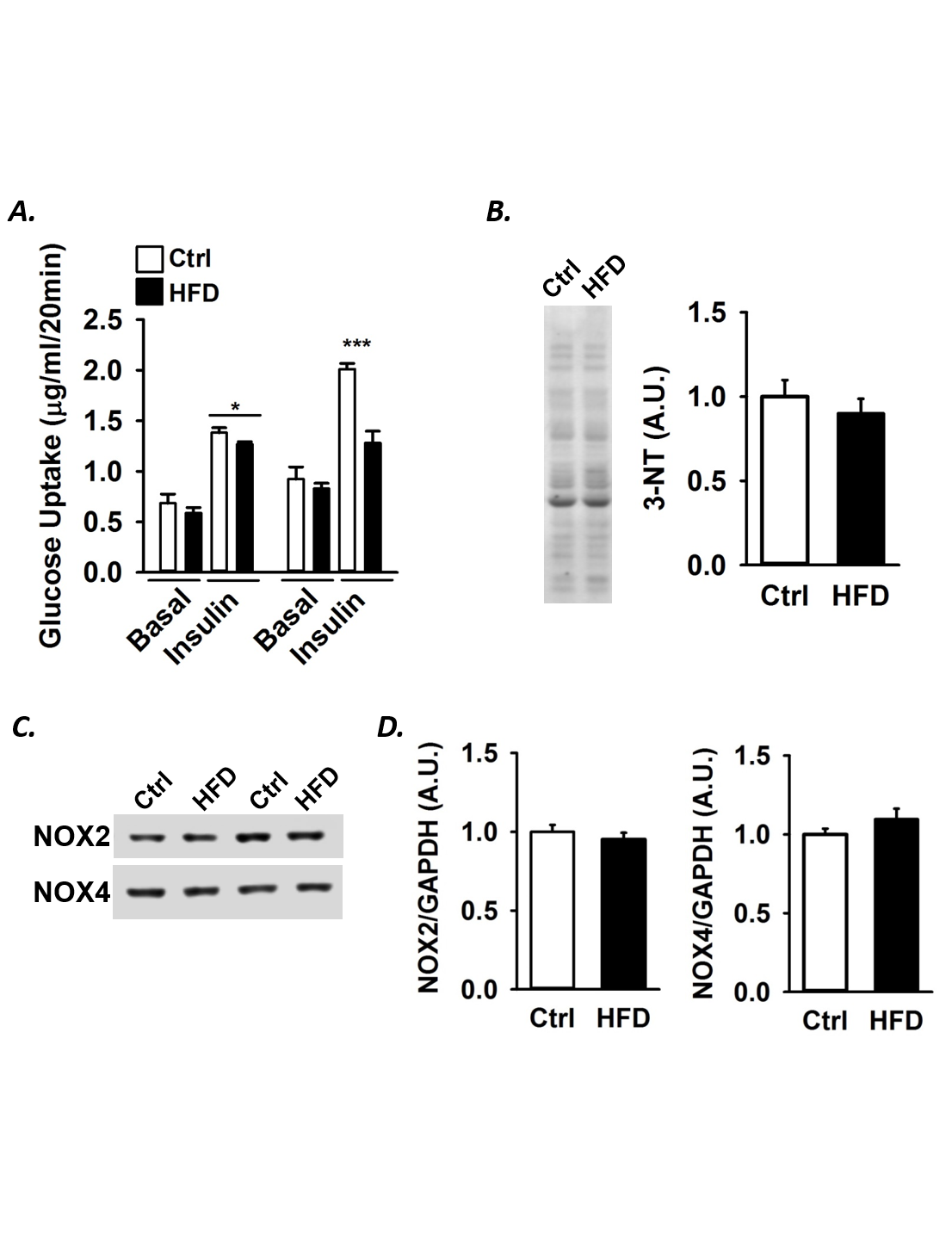

Supplement: S1 Fig — A. Glucose uptake in fast-twitch (EDL) and slow-twitch (soleus) muscles shows that mice on fat diet have a decreased insulin-mediated glucose uptake, which verifies a systemic insulin resistance (n = 6). Data are mean ± SEM; * P < 0.05, ***P <0.001 vs. control). B. Representative Western blots and mean data ± SEM (n = 6) of protein nitration (anti-3-nitrotyrosine (3-NT; 1:500, Abcam)) in left ventricles from control mice and HFD mice. Representative Western blots (C) and mean data ± SEM (D, n = 6) of total expression of NOX2/gp91phox (1:1000, Abcam) and NOX4 (1:2000, Thermo-Pierce) in left ventricles from control mice and HFD mice. (TIF) [file pone.0167090.s001.tif]
